# Are lecanemab and donanemab disease‐modifying therapies?

**DOI:** 10.1002/alz.14114

**Published:** 2024-08-03

**Authors:** Timothy Daly, Kasper P. Kepp, Bruno P. Imbimbo

**Affiliations:** ^1^ Bioethics Program FLACSO Argentina Tucumán Buenos Aires Argentina; ^2^ Science Norms Democracy UMR 8011 Sorbonne University Paris France; ^3^ Section of Biophysical and Biomedicinal Chemistry Kongens Lyngby Denmark; ^4^ Research & Development Chiesi Farmaceutici Parma Italy

Encouraging results from two large 18‐month, double‐blind, placebo‐controlled studies of the anti‐amyloid beta (Aβ) monoclonal antibodies (mAb) lecanemab^1^ and donanemab^2^ in early Alzheimer's disease (AD) have motivated claims that they are disease‐modifying. Indeed, these trials dramatically reduced brain Aβ‐positron emission tomography (PET) burden and demonstrated a highly significant, albeit clinically modest, delay of cognitive decline. We define disease‐modifying as a causal intervention with a corresponding long‐term benefit, rather than short‐term symptomatic improvement observed with previously approved drugs affecting cholinergic neurotransmission. We discuss two testable criteria for disease modification and whether these mAbs have fulfilled them.

## 1 CRITERION 1: CLINICAL EFFECT–BIOMARKER CORRELATION

One criterion is a consistent relationship between clinical efficacy and a disease‐specific biomarker (Aβ‐PET load) in the same trial.^3^ Lecanemab and donanemab apparently satisfy this requirement, consistent with the most recent formulation of the amyloid hypothesis, predicting that steep removal of amyloid would lead to delayed clinical impact.^4^ However, the correlation between Aβ‐PET and cognitive and clinical performance has only been demonstrated on aggregated treatment group data, but not at the individual patient level. At least 30% of elderly people have significant brain amyloid load without cognitive symptoms, and other trials with anti‐Aβ mAb (eg, recently gantenerumab) showed no beneficial effects despite significantly decreased Aβ‐PET. We thus do not know whether the aggregate correlation arises from Simpson's paradox where the strongest reduction in Aβ‐PET also means the least clinical effect, and vice versa. Meta‐analyses offer “inconsistent evidence” on the strength of association between Aβ‐PET reduction “and several cognitive rating scales for Alzheimer's disease.”^5^

For a complex disease like AD, a single key biomarker may not fully explain the cognitive and clinical effects of a drug. Paradoxically, in the case of aducanumab, the exact opposite argument was proposed by the US Food and Drug Administration (FDA), that is, that a biomarker by itself defines clinical effect as “reasonably likely.”^6^ Thus, there is a valid debate on whether biomarker correlation is necessary for disease modification.^7^ However, if used as an argument for disease modification as in the present case, evidence would involve a correlation shown using the individual patient data, given the aforementioned issues of aggregate data.

**RESEARCH IN CONTEXT**

1. **Review**: Lecanemab and donanemab provide hope for the treatment of Alzheimer's disease (AD), since in 18‐month controlled studies, both drugs dramatically reduced brain amyloid plaques in AD patients and produced highly statistically significant but modest cognitive and clinical benefits versus placebo.
2. **Interpretation**: Both drugs come with significant adverse events and risks of biases in the efficacy estimates with the caregiver input. If the drugs are “disease modifying,” long‐term benefits could motivate administration, but the definitions and criteria to establish such disease modification vary.
3. **Future directions**: We discuss the main criteria for disease modification and conclude that there is not strong evidence yet for claiming a modification of the natural history of the disease by these drugs, but we describe experiments that could provide this evidence.

## 2 CRITERION 2: PROGRESSIVE, OBJECTIVE IMPROVEMENTS IN COGNITION

Another criterion for disease modification is that clinical benefits accumulate over a longer time, arguably also after ending treatment. Amyloid buildup presumably takes many years, and once removed, would arguably require minimal further treatment to balance the small amounts of new amyloid formed. The absolute effect size (Cohen's *d* coefficient) at 18 months for the scale measuring the clinical performance of participants (Clinical Dementia Rate‐Sum of Boxes, CDR) is 0.21 in the lecanemab study and 0.23 in the donanemab study,^8^ that is, small,^9^ and similar to symptomatic drugs. The claim that a more robust effect size takes a longer time to mature would require defining time cutoffs where drug superiority versus symptomatic drugs is predicted, in order to be scientifically falsifiable and to make disease modification clinically relevant to patients with limited life span.

The separation of efficacy curves between a disease‐modifying treatment and placebo group should manifest over a longer time in fully objective cognitive scales less sensitive to unblinding bias. While informant‐dependent scales are very clinically relevant at a given time, slope divergence for an objective scale over time would provide strong evidence of disease modification, suggestive of continuous benefit over the disease course. Statistically, the divergence of the terminal slopes of the efficacy variable can be tested, with statistical significance of the interaction of the “treatment” and “time” factors in the appropriate analysis of the covariance model suggesting disease modification. Testing such an effect on the terminal data points of the CDR‐SB and other variables should be a priority.

**FIGURE 1:**
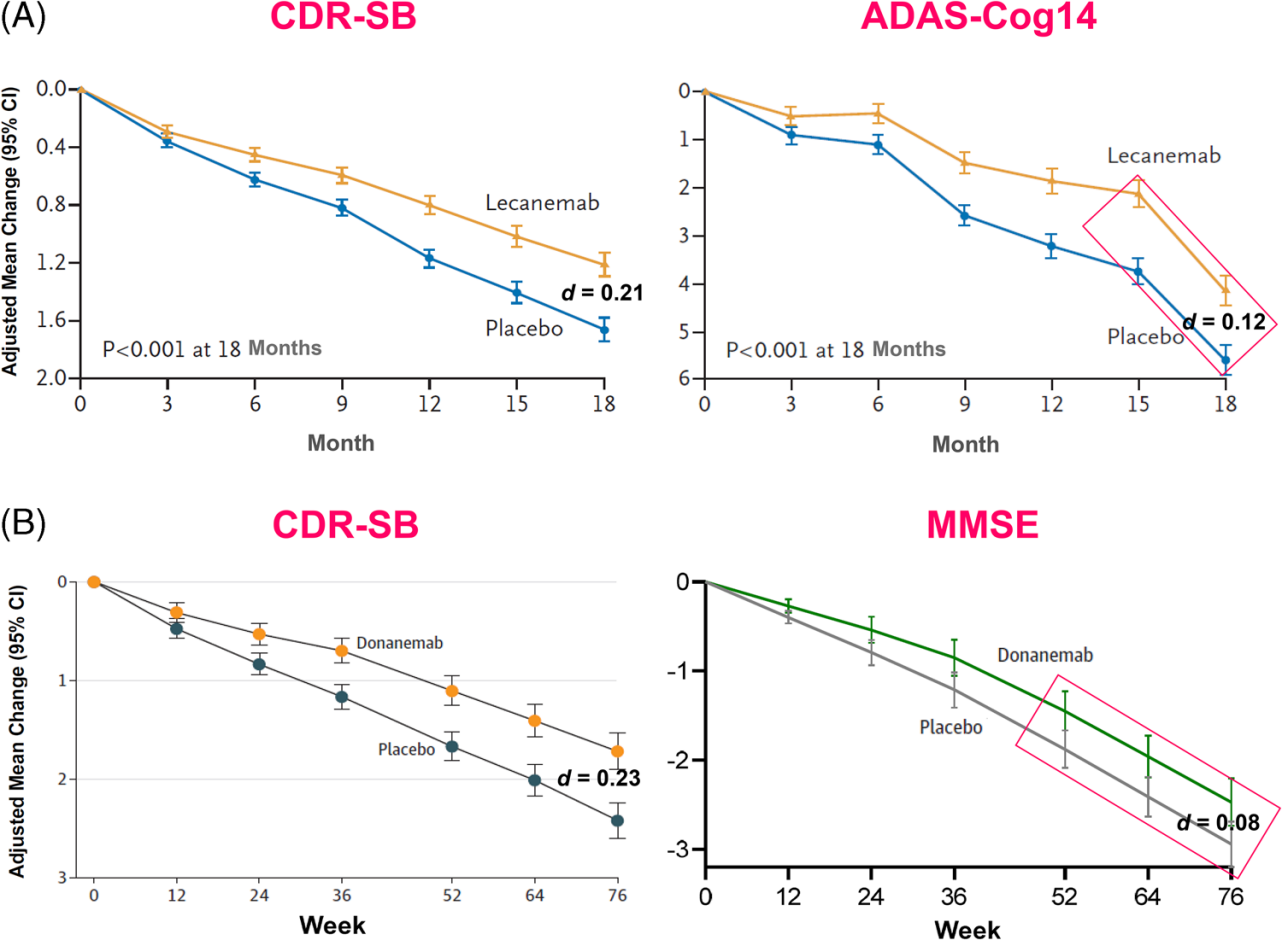
Examining the cognitive impact of recent antibodies for Alzheimer's disease (AD). (A) Mean Clinical Dementia Rating–Sum of Boxes (CDR–SB) and ADAS‐Cog profiles versus time in the lecanemab and placebo groups of the Clarity AD study (modified from van Dyck et al., 2023). ADAS‐Cog14: 14‐item cognitive subscale of the Alzheimer's Disease Assessment Scale. (B) Mean CDR‐SB and Mini‐Mental State Examination (MMSE) profiles versus time in donanemab and placebo groups of TRAILBLAZER‐ALZ 2 study (modified from Sims et al., 2023). *d*: Cohen's coefficient (effect size).

A visual inspection of both trial results (Figure 1) suggests slope divergence of the terminal CDR‐SB mean values. For a more objective cognitive measure of efficacy (cognitive subscale of the Alzheimer's Disease Assessment Scale [ADAS‐Cog] and Mini‐Mental State Examination [MMSE]), this divergence is not observed for lecanemab (Figure 1A, right panel) or donanemab (Figure 1B, right panel). Lecanemab's effect size on the ADAS‐Cog14 is almost two times less than on CDR‐SB (Figure 1A), and donanemab's effect on MMSE is almost three times less than on CDR‐SB (Figure 1B). It is reasonable to hypothesize that the “treatment by time” interaction is not significant for both ADAS‐Cog and MMSE. The lecanemab slopes of the Alzheimer's Disease COMposite scale (four cognitive items of the ADAS‐Cog plus two cognitive items of the MMSE, plus all six items of the CDR‐SB) also appear parallel (data not shown^1^). This suggests that the objective cognitive measures, when combined with the CDR‐SB, are responsible for the lack of divergence of the CDR‐SB slopes. Interestingly, the Alzheimer's Disease Cooperative Study ‐ Activities of Daily Living for Mild Cognitive Impairment (ADCS–MCI‐ADL) functional scale slopes diverge, confirming that the slopes of the scales based on caregiver input diverge, while the slopes of the objective cognitive variables collected by the patients are parallel. A more precise estimate of the slopes of the efficacy variables could be obtained by extending the observation period of patients to 24 or 36 months, but this would inevitably lead to an increase in the number of already‐high drop‐outs with an increase of missing data and unpredictable biases.

Delayed‐start designs could provide a higher level of evidence for disease modification in AD,^7^ as in Parkinson's disease.^10^ Participants would be randomized to receive either placebo or active treatment during stage one, and during stage two, all would receive active treatment. Under disease modification, the delayed‐start placebo group would be expected to never catch up to the early‐start active treatment group, though such trials may be invalidated by differential drop‐out rates during stage one.^11^

## 3 CONCLUSION: DESIRABILITY OF ANTI‐AMYLOID TREATMENTS

To achieve disease modification, we must measure efficacy over time while controlling for confounders. Available data do not confirm that lecanemab and donanemab are disease‐modifying. Adverse effects, including symptomatic amyloid‐related imaging abnormalities (ARIA), may also contribute to unblinding that could inflate efficacy, occurring in 21.5% of lecanemab (vs 9.5% on placebo) and in 36.8% of donanemab patients (vs 14.9% on placebo).^12^ Extending the observation period to 24 or 36 months would provide a more precise estimate of efficacy slopes but would lead to more drop‐outs (already high at 18 months), missing data, and unpredictable biases. Although autosomal dominant early‐onset AD would provide the paradigmatic test of early disease modification,^13^ the small number of these cases would complicate efforts to obtain statistical power. Thus, ongoing prevention trials in cognitively normal subjects at risk of developing AD with lecanemab (AHEAD3 and AHEAD45 studies) and donanemab (TRAILBLAZER‐ALZ 3 study) will be crucial for verifying disease modification and for assessing risk–benefit profiles of amyloid‐lowering treatments.^14^ Finally, even if disease modification is proven, benefits must be “noticeable, valuable, and worthwhile in the context of costs and risks”^15^ for these drugs to have their hoped‐for therapeutic impact.

## CONFLICT OF INTEREST STATEMENT

Drs. Timothy Daly and Kasper Planeta Kepp have no conflicts of interest to declare. Dr. Bruno P. Imbimbo is an employee at Chiesi Farmaceutici. He is listed as an inventor in a number of Chiesi Farmaceutici's patents of anti‐Alzheimer drugs. Author disclosures are available in the supporting information.

## Supporting information

Supporting Information
